# Formulation and Characterization of Gum Arabic Stabilized Red Rice Extract Nanoemulsion

**DOI:** 10.3390/polym14101938

**Published:** 2022-05-10

**Authors:** Aarti Bains, Agnieszka Najda, Prince Chawla, Joanna Klepacka, Sanju Bala Dhull, Pardeep Kumar Sadh, Mohammed Azhar Khan, Ravinder Kaushik

**Affiliations:** 1 Department of Biotechnology, CT Institute of Pharmaceutical Sciences, South Campus, Jalandhar 144020, Punjab, India; aarti05888@gmail.com; 2 School of Biotechnology, Shoolini University, Solan 173229, Himachal Pradesh, India; mk.azhar1@gmail.com; 3 Department of Vegetable and Herbal Crops, University of Life Science in Lublin, Doświadczalna Street 51A, 20280 Lublin, Poland; 4 Department of Food Technology and Nutrition, Lovely Professional University, Phagwara 144411, Punjab, India; 5 Department of Commodity Science and Food Analysis, Faculty of Food Science, University of Warmia and Mazury in Olsztyn, Oczapowskiego 2, 10719 Olsztyn, Poland; klepak@uwm.edu.pl; 6 Department of Food Science and Technology, Chaudhary Devi Lal University, Sirsa 125055, Haryana, India; sanjudhull@gmail.com; 7 Department of Biotechnology, Chaudhary Devi Lal University, Sirsa 125055, Haryana, India; pardeep.sadh@gmail.com; 8 School of Health Sciences, University of Petroleum and Energy Studies, Dehradun 248007, Uttrakhand, India; ravinder_foodtech2007@rediffmail.com

**Keywords:** nanoemulsion, *Oryza sativa* L., anti-inflammatory, zeta potential, gum arabic

## Abstract

Interest in the utilization of plant-based bioactive compounds in foods has increased due to their biochemical activities and as alternatives in the reduction of high concentrations of chemical utilization. However, some of these additives are hydrophobic, thus being harder to disperse into the hydrophilic food matrix. Therefore, an oil-in-water nanoemulsion (RRE1-RRE10) was formulated with different concentrations of red rice extract (1–10% *w*/*v*). Nanoemulsion showed droplet sizes within the range of 157.33–229.71 nm and the best formulation (RRE5) was selected based on the creaming index which was stable to flocculation over a range of temperatures (30–90 °C), pH (2–9), and salt concentration (100–600 mM). It showed significantly improved antioxidant and anti-inflammatory activity as compared to its other counterparts. Potential antimicrobial activity against *Staphylococcus aureus* was attributed to RRE5 nanoemulsion as compared to *Escherichia coli*. Therefore, due to the potential bioactivity of RRE5 nanoemulsion, it can be scaled up at the industrial level.

## 1. Introduction

Rice, a rich source of bioactive constituents (phenolic components and minerals), contributes to 25% of the world’s calorific intake [1]. However, the world’s population consumes white rice most commonly, and an interest among consumers is increasing in the intake of pigmented rice [2]. The presence of anthocyanins (cyanidin-3-*O*-glucoside, peonidin-3-*O*-glucoside, cyanidin-3-*O*-rutinoside, and cyanidin-3-*O*-galactoside) and proanthocyanidins (a class of polymeric phenolic compounds consisting mainly of flavon-3-ol units i.e., catechin, epicatechin, and their 3-*O*-gallates and epigallates) in various layers of the pericarp, seed coat, and aleurone give the colored appearance of pigmented rice [1]. In addition, gallic, protocatechuic, hydroxybenzoic, p-coumaric, ferulic, sinapic acid, cyanidin-3-*O*-glucoside, peonidin-3-*O*-glucoside, flavan-3-ol (+) catechin and (−) epicatechin, flavonols, isoflavones, γ-oryzanol, steryl, triterpene alcohol ferulates proportions, and tocopherols are the principal components of the red rice. These bioactive compounds have the potential for human health and can act as anti-tumor, anti-atherosclerosis, anti-diabetic, anti-allergic agents, alleviating gallstones, anticancer activity, and anti-inflammatory effects [3,4,5]. In recent years, chronic inflammatory diseases have been the primary cause of death in the world. The World Health Organization (WHO) ranks chronic diseases as the greatest threat to human health [6]. Inflammation is usually stated as a complex biological reaction of vascular tissues to harmful stimuli. In addition, inflammation is combined with ache, and it includes an intensification of protein denaturation, membrane variation, and an increase in vascular absorptivity [7]. Moreover, inflammation is defined as the reaction of the human body to deactivate annexing stimuli, eliminate the pains, and set the phase for tissue repair and the process is augmented by the excretion of chemical intermediaries from injured cells or tissues and migrating cells [8]. Although inflammation is a body defensive phenomenon, insufficient regulation and unsuitable as in disparity to self-tissue, it could be the reason for severe diseases and injuries [9]. To combat inflammation, steroidal and non-steroidal drugs (NSAIDs) are most frequently used. However, these allopathic medicines have numerous adverse effects, such as gastric ulcers and tissue irritations [8,10]. Therefore, natural bioactive components and phytochemicals with anti-inflammatory activity have gained great interest in recent years. Wheat and rice are a staple food across the world and people are more dependent upon cereals in terms of nutrition [4]. In addition, microorganisms are becoming more resistant to standard antibiotic drugs; therefore, researchers are showing interest in plant extract-based antimicrobial components [11]. Several reports have been published on the effective use of plant extracts as antimicrobial agents [4,11,12,13,14]. Both Gram-positive and Gram-negative bacteria have different sensitivity toward antimicrobial agents, therefore, plant extracts could be a better approach in terms of antimicrobial efficacy [11,14]. Moreover, the bioactive constituents of plant extracts are more prone to environmental and oxidative stress and these hydrophobic components have the least solubility in water [15,16]. Therefore, to attain the significant features of rice extract, it is required to increase the water solubility; thus, emulsification is a remarkable process that is frequently used to enhance the functionality of the bioactive component [17,18,19]. Furthermore, several in vitro and in vivo reports on emulsification revealed significantly improved stability and bioavailability of the bioactive components which is achieved through the formation of mixed micelles in the small intestine and that are swiftly and completely absorbed by the epithelium cells [20]. In the case of antimicrobial properties, nanoemulsion showed significantly higher antimicrobial efficacy in comparison with plant extracts [21]. Furthermore, gum arabic is a neutral or slightly acidic, polysaccharide complex, containing about 2% of the polypeptide. The branches mainly consist of 1,3-linked β-D-galactopyranosyl units with 1,6-linked β-D-galactopyranosyl side chains to which there are linked many arabinosyl, uronic acid, and rhamnose residues, where an approximately 43 amino acid residue peptide sequence is supposed to be buried. In addition, three main fractions, i.e., arabinogalactan-peptide, arabinogalactan-protein, and glycoprotein, have been isolated by hydrophobic interaction chromatography [22]. It has also been revealed that gum arabic has excellent emulsifying properties due to CH...π interactions (this is an attractive interaction between a hydrogen atom from a molecule or a molecular fragment X–H in which X is more electronegative than H, and an atom or a group of atoms in the same or a different molecule, in which there is evidence of bond formation) [23]. For the formulation of stable nanoemulsion, a suitable emulsifier plays a vital role; therefore, gum arabic was used as a biopolymer for the fabrication of nanoemulsion [24,25]. Furthermore, ultrasonication (a high-energy technique) is a frequently used technique for the synthesis of stable oil in water nanoemulsion [26]. For the preparation of rice extract nanoemulsion, the oil phase is required; hence, rice bran oil was used for the dissolution of red rice extract and formulation of the continuous phase of the nanoemulsion. Only a few reports have been published on the formulation of plant extract nanoemulsion and no data are available for the formulation of red rice extract nanoemulsion. Therefore, the present study was carried out based on the following objectives: (i) Formulation and characterization of the gum arabic stabilized red rice extract nanoemulsion, (ii) effect of storage on the antimicrobial efficacy of the nanoemulsion, and (iii) oxidative stability and anti-inflammatory activity of the selected nanoemulsion.

## 2. Materials and Methods

### 2.1. Materials

Red rice variety ‘Ram Jawain’ was obtained from the farms of district Kangra, Himachal Pradesh, India. Taxonomical identification was confirmed by the Department of Botany, Shoolini University, Solan Himachal Pradesh, India. Deve Herbes pure rice bran oil was procured from the local market of Solan, Himachal Pradesh, India.

#### Chemicals

L-ascorbic acid, bovine serum albumin, 2-2-diphenyl-1-picrylhydrazyl, gum arabic, 2-thiobarbituric acid, phosphate buffer saline (PBS), and cyclohexanone were purchased from Sigma Aldrich Co. St. Louis, MO, USA. Sodium hydroxide, ethanol, citric acid, hydrochloric acid, nitric acid, Tween 80, phosphate buffer, and ammonium sulfate were purchased from Loba Chemie Pvt Ltd. Navi Mumbai, India. Standard antibiotics (ciprofloxacin), Muller Hinton Agar, nutrient agar, and nutrient broth were procured from Hi-Media Private Limited, Mumbai, India. Gram-positive and Gram-negative bacterial strains, i.e., *Staphylococcus aureus* (MTCC 3160) and *Escherichia coli* (MTCC 443), were obtained from the Microbial Type Culture Collection (MTCC), Institute of Microbial Technology, Chandigarh, India. Analytical grade chemicals and acid-washed glassware were used throughout the experiments.

### 2.2. Methods

#### 2.2.1. Extraction of Ethanolic Extract from Red Rice

Red rice kernel was washed properly with triple distilled water and dried in a hot air oven (NSW-142, Narang Scientific Works, Ambala, India) at 30 °C. Dried red rice was powdered using a mechanical grinder (Braun AG Frankfurt A.M. Mx 32, Germany). Ethanolic extract of red rice was prepared by following the literature [27]. Briefly, 50 g powdered red rice was dispersed in 500 mL (1:10 *w*/*v* ratio) of absolute ethanol in a conical flask and kept in an orbital shaker (MaxQ 4000, Thermofisher Scientific Pvt. Ltd., Mumbai, India) for 72 h. For the evaporation of the solvent, a modified solvent evaporation technique was employed, and the sample was then filtered through Whatman filter paper no. 1. Evaporation of the solvent was done at refrigerated temperature (4–7 °C) for 72 h and the dried extract (383 mg/g rice powder) was then stored at −20 °C in airtight glass tubes for further analysis.

#### 2.2.2. Preparation of Oil in Water Nanoemulsion

The nanoemulsion was prepared by following the method proposed by [28]. Dried red rice extract (RRE) with a mass of 1, 3, 5, 7, and 10 g was added to 10 mL of rice bran oil and further mixed using a magnetic stirrer (SPINOT MC 02, Tarsons, Kolkata, India) for 2 h. The mixture of oil and RRE was then added to the aqueous phase (1% *w*/*v* gum arabic and 250 µL tween 80) solutions and mixed for 15 min. The whole blend was then subjected to ultra-sonication for the synthesis of RRE nanoemulsion using a probe sonicator (Sonics and Materials Inc., New Town, CT, USA) at 5 °C with a 5.0-s pulse rate for 20 min. The beaker containing the sample was kept in an ice bucket to control the rise in temperature during the sonication process. Nanoemulsion without red rice extracted was formulated and used as a control.

#### 2.2.3. Creaming Index for the Selection of Suitable Nanoemulsion

For the selection of suitable nanoemulsion sample creaming stability of the nanoemulsion, samples were determined by following the method described by [15]. Briefly, glass bottles filled with 50 mL nanoemulsion were stored at accelerated conditions (80 °C) for 15 days. Then, the nanoemulsion samples were evaluated for physical stability by the percentage of creaming.

The creaming index was determined using the following formula:

Creaming index (%) = (volume of the cream layer after heating)/(volume of the emulsified layer) × 100(1)

##### Droplet Size Distribution and Zeta Potential of the Nanoemulsion

The dynamic light scattering technique was used for the determination of droplet size. Briefly, 1% (*v*/*v*) nanoemulsion was prepared by diluting nanoemulsion in 0.05 M phosphate buffer (pH 7). Zeta potential analyzer (Zetasizer Nano ZS, Malvern Instruments Ltd., Malvern, UK) was used for the determination of droplet size and zeta potential of the nanoemulsion.

##### Scanning Electron Microscopy of the Nanoemulsion

The selected nanoemulsion was subjected to morphological evaluation using Scanning electron microscopy (JEOL JSM-6510 LV ICA, Peabody, MA, USA). Briefly, 1% osmium tetraoxide in double-distilled water was used to fix the few droplets of selected nanoemulsion. After stabilizing the fixed RRE5 nanoemulsion, the sample was then centrifuged at 10,000× *g* for 20 min followed by rinsing with triple distilled water. The process was repeated thrice and the top particle layer was removed. The washed sample was then stabilized for 20 min before SEM analysis.

##### Emulsion Stability Testing

Freshly formulated selected nanoemulsion was exposed to a series of environmental strains that might come across in commercial applications and then stored for 24 h at 30 °C temperature before analysis. Variation in droplet size and zeta potential confirmed the stability of the selected nanoemulsion.

##### Effect of Thermal Processing

Freshly formulated nanoemulsion was transferred into glass test tubes and these were then incubated in a water bath (NSW-125, Narang Scientific Works, Ambala, India) set at temperatures ranging from 30, 45, 60, 75, and 90 °C for 30 min, and then cooled to room temperature (27 °C).

##### Effect of pH

Freshly formulated nanoemulsion samples were transferred into 25 mL glass beakers and adjusted to different pH values (2.0–9.0) using 0.1 N NaOH and 0.1 N HCl solutions.

##### Ionic Strength

Variation in droplet size and zeta potential confirmed the ionic strength of nanoemulsion after the addition of different concentrations of sodium chloride (100, 200, 400, 600 mM).

### 2.3. Thiobarbituric Acid (TBA) Value of Nanoemulsion

The TBA value of nanoemulsion was determined by following the method proposed by Sharma et al., 2017 [29]. Briefly, the nanoemulsion (5 mL) sample was mixed with a freshly prepared 0.025 M TBA (neutralized with sodium hydroxide and 2 M citric acid) reagent. The mixture of nanoemulsion and TBA reagent was heated instantaneously in a boiling water bath for 10 min. Further, 10 mL cyclohexanone and 1 mL of 4 M ammonium sulfate were added to the mixture and centrifuged at 5000× *g* for 5 min at room temperature (27 °C). The orange-red colored cyclohexanone supernatant was collected and absorbance was measured at 532 nm using a UV–Visible spectrophotometer (Evolution 201, Thermo Scientific, Mumbai, India).

### 2.4. Antioxidant Assay

#### DPPH (2,2-Diphenyl-1-picrylhydrazyl) Radical Scavenging of Nanoemulsion

Antioxidant efficacy was estimated using the method proposed by Sadh et al., 2018 [27]. Selected nanoemulsion and red rice extract were stored for 30 days at 30 °C and subjected to antioxidant activity at a regular interval of 5 days. Briefly, 0.1 mM DPPH was prepared and an aliquot of RRE nanoemulsion (200 µL) was added to 2 mL of prepared DPPH solution and incubated for 30 min in dark. Change in DPPH color was measured at 517 nm using UV-spectrophotometer. The percentage of DPPH radical scavenging activity (RSA) of nanoemulsion was determined using the following formula:

Inhibition (%) = ((AD − AS)/AD) × 100(2)

where AD is the absorbance of the DPPH and AS is the absorbance of the sample.

### 2.5. Anti-Inflammatory Assay

#### 2.5.1. Protein (BSA) Denaturation Assay

This assay was performed according to the method proposed by Gunathilake et al., 2018 [10]. Briefly, 200 µL RRE nanoemulsion was properly mixed with 1% BSA (200 µL) and PBS (4.78 mL, pH 6.4), and then the mixture was incubated in an incubator (37 °C) for 15 min. After incubation, the reaction mixture was heated at 70 °C for 5 min and immediately cooled at room temperature (27 °C). After cooling, the turbidity of the reaction mixture was measured at 660 nm using a UV–Visible spectrophotometer. PBS was taken as control and percentage inhibition of protein denaturation was determined by using the following formula:

Inhibition of denaturation (%) = (1 − AC/AT) × 100(3)

where AC is the absorbance of the control sample and AT is the absorbance of the test sample.

#### 2.5.2. HRBC Membrane Stabilization Assay

In this assay, blood (5 mL) was collected from a healthy human volunteer (the research project was approved by the institutional ethical committee (IEC), Shoolini University, Solan, Himachal Pradesh, with the IEC number SUIEC/19/25) who did not take NSAIDs (nonsteroidal anti-inflammatory drugs) for 2 weeks. The blood was dissolved in an equal volume of sterilized Alsever solution (20.5 g Dextrose, 8 g sodium citrate, 0.55 g citric acid, and 4.2 g sodium chloride in 1000 mL water) and was then centrifuged at 3000× *g* for 15 min. Packed cells obtained after centrifugation were washed with isosaline. The assay mixture that contains 500 µL RRE nanoemulsion, 0.15 M (1 mL) phosphate buffer with pH 7.4, 0.36% (2 mL) hyposaline solution, and 500 µL HRBC suspension was prepared. This assay mixture was then incubated in the BOD incubator at 37 °C for 30 min and centrifuged at 3000× *g* for 20 min. Diclofenac sodium and distilled water were used as positive and negative control, respectively. The supernatant containing the content of hemoglobin was estimated by measuring the absorbance at 560 nm using a UV–Visible spectrophotometer [12]. Anti-inflammatory activity was calculated as follows:

Protection (%) = 100 − [ODs/ODc × 100](4)

Here, ODs is the optical density of the sample and ODc is the optical density of the control.

### 2.6. Antimicrobial Efficiency of Nanoemulsion

The in vitro antimicrobial activity of red rice extract and nanoemulsion was evaluated against pathogenic Gram-positive and Gram-negative bacteria, namely *Staphylococcus aureus* and *E. coli*, respectively. The antimicrobial assay was performed using the agar well diffusion method proposed by Bains and Chawla (2020) [30]. Herein, Muller Hinton agar enriched with 4% NaCl was used to inoculate bacterial strain (1.5× 108 cells/mL). The wells were made on plates using cork borer wells. For stock solution, the red rice extract (10 mg) and nanoemulsion (1000 µL) were dissolved in 10 mL of DMSO (5%) separately and each sample (50 µL) was poured into different agar wells with the help of a micropipette. Commercially available antibiotic, chloramphenicol (1 µg/mL) was used as positive control and DMSO was used as a negative control. The MHA plates were inoculated with pathogenic bacterial strains and incubated at 37 °C for 24 h. Results of antimicrobial activity of both red rice extract and nanoemulsion were measured as the zone of inhibition in mm.

#### Time-Kill Study

A time-kill study was performed by the method followed by Chawla et al., 2020 [31]. Herein, 100 µL of both red rice extract and nanoemulsion solution was used for all pathogenic microbial strains. Aliquots of 100 µL were taken from each sample after 0, 2, 4, 6, 8, 10, 12, 24, 36, 48 h intervals and were serially diluted and spread on MHA plates followed by incubation at 37 °C for 24 h. Results were obtained after calculating Log CFU/mL for each sample.

### 2.7. Statistical Analysis

Statistical analysis was done by following the method proposed by Kaushik et al., 2018 [32]. A significant difference between the samples was confirmed by one-way analysis of variance (ANOVA) and the comparison between means was calculated by critical difference (CD value). Microsoft Excel, 2016 (Microsoft Corp., Redmond, WA, USA) was used for the calculation of means and standard error mean.

## 3. Results and Discussion

### 3.1. Formation of Gum Arabic Stabilized Nanoemulsion

**Figure 1:**
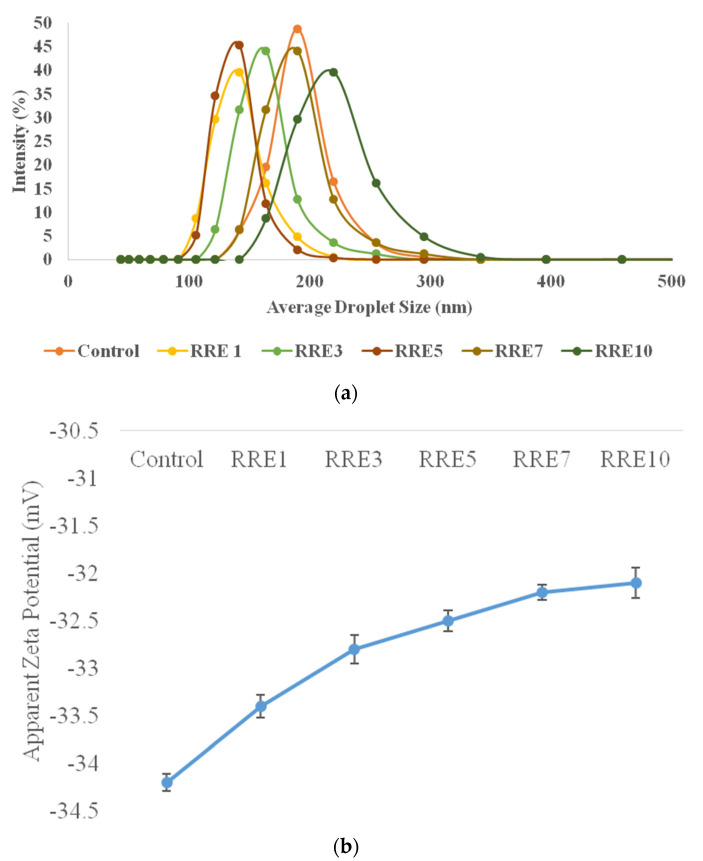
(**a**) Average droplet size, and (**b**) apparent zeta potential of control, RRE1, RRE3, RRE5, RRE7, and RRE10 nanoemulsion. Data are presented as means ± SD (*n* = 3).

Initially, the influence of red rice extract concentration on the formation of oil-in-water nanoemulsion using a fixed concentration of gum arabic (1%) was investigated. Therefore, control, RRE1, RRE3, RRE5, RRE7, and RRE10 nanoemulsion were formulated and results of average droplet size are depicted in Figure 1a. The average particle size of all the nanoemulsion samples ranged from 157.33 to 229.71 nm. RRE10 nanoemulsion samples showed significantly (*p* < 0.05) higher droplet size as compared to other nanoemulsion samples, however, RRE1, RRE3, and RRE5 showed non-significant (*p* < 0.05) differences from each other. Furthermore, no sign of flocculation or creaming was observed even at a higher concentration of rice extract. Gum arabic extensively altered the charge dispersal of surface and interfacial viscosity of the emulsion system directly affects emulsion behavior in terms of colloidal stability, absorption, and bioavailability. Increasing the concentration of extract could be the possible reason for significantly increased droplet size.

Moreover, the zeta potential of all the emulsion samples is depicted in Figure 1b. The zeta potential dimensions of all nanoemulsion samples ranged from −33.4 to −32.1 mV. Results from the figure revealed a non-significant (*p* < 0.05) difference in zeta potential values of all the emulsion samples. In addition, increasing the concentration of rice extract did not influence the charge distribution of all nanoemulsion samples. Negative charge distribution on nanoemulsion samples was due to the prearrangement of gum arabic with rice bran oil, Tween 80, and rice extract through hydrophobic binding sites and anionic interactions. Results are well supported by the literature results provided by Khan et al., 2013 and Yao et al., 2018 [33,34], who revealed particular interaction of gum arabic with continuous phase and surfactants on total charge distribution of nanoemulsion.

### 3.2. Creaming Index of Nanoemulsion

**Figure 2:**
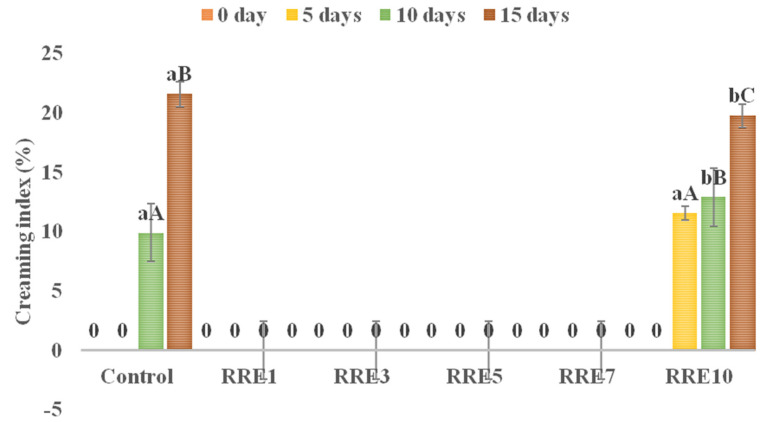
Creaming index of control and RRE nanoemulsions during storage of 15 days. Data are presented as means ± SD (*n* = 3). ^a–b^ Means within the column with different lowercase superscripts are significantly different (*p* < 0.05) from each other. ^A–C^ Means within the column with different uppercase superscripts are significantly different (*p* < 0.05) from each other.

The effect of rice concentration on the creaming stability of all control and RRE nanoemulsion samples is depicted in Figure 2. The control sample showed a significantly (*p* < 0.05) higher creaming index as compared to RRE nanoemulsion samples. For the control sample, a visual creaming appeared on the 10th day of storage. With increasing concentration of rice extract nanoemulsion, RRE1–RRE7 did not exhibit a visual creaming process on the 15th day of storage, however, RRE10 showed 11.56 on the 5th day, and 12.89 on the 10th day, and 19.74% creaming index on the 15th day. Furthermore, RRE10 showed a significant (*p* < 0.05) difference in the creaming index with an increasing concentration of rice extract.

The stability of nanoemulsion to coalescence and flocculation depends upon droplet size and interfacial surface tension between the aqueous and continuous phases. As a consequence of coalescence in RRE10 nanoemulsion, the droplet size distribution tends to be enlarged and moves towards larger sizes, eventually favoring creaming and destabilization of the emulsion. Among the most important effects relevant for the hindering of droplet coalescence, it is worth mentioning the repulsive interaction between adsorbed layers, the interfacial coverage, the steric effects, and the high dilational viscoelasticity of the interfacial layers. The repulsive interactions are particularly relevant when ionic surfactants are concerned. Hence, the RRE10 nanoemulsion did not exert smaller droplets due to weak attractive forces than that of the repulsive forces [19]. Furthermore, according to previously published reports, if nanoemulsion keep standing for several hours, they would phase separate into a cream emulsion layer and a serum layer despite the initial small droplet sizes. Measurements of droplet sizes in the cream layer suggest that droplets attained very large diameters without flocculating. Since no coalescence in emulsions with polyphenolic extract contents was observed, the emulsions likely destabilized due to Ostwald ripening [31]. However, if a critical concentration is exceeded, this “retardation” effect is lost and the emulsion droplets rapidly grow in size. Under the ultrasonication condition, where local temperatures increase due to cavitation, such processes could be accelerated leading to the observed increases in droplet diameters [35]. Based on the results, a hypothetical model of red rice extract nanoemulsion was proposed, where the hydrophilic part of gum arabic protrudes into the aqueous phase and the hydrophobic part remains towards red rice extract molecules. Intense high pressure and cavitations in the sonication process during emulsification might induce chemical deformation of the oil phase, while RRE1 to RRE5 nanoemulsion samples were appreciably stable due to substantially mono-dispersed, small-sized, and strong repulsion between red rice extract nanodroplets, adequately coated by gum arabic. As discussed in the previous section, RRE1 to RRE5 nanoemulsion samples showed a non-significant difference in droplet size; therefore, stable RRE5 nanoemulsion containing maximum concentration of red rice extract was selected for further analysis.

### 3.3. Morphology of Nanoemulsion

**Figure 3:**
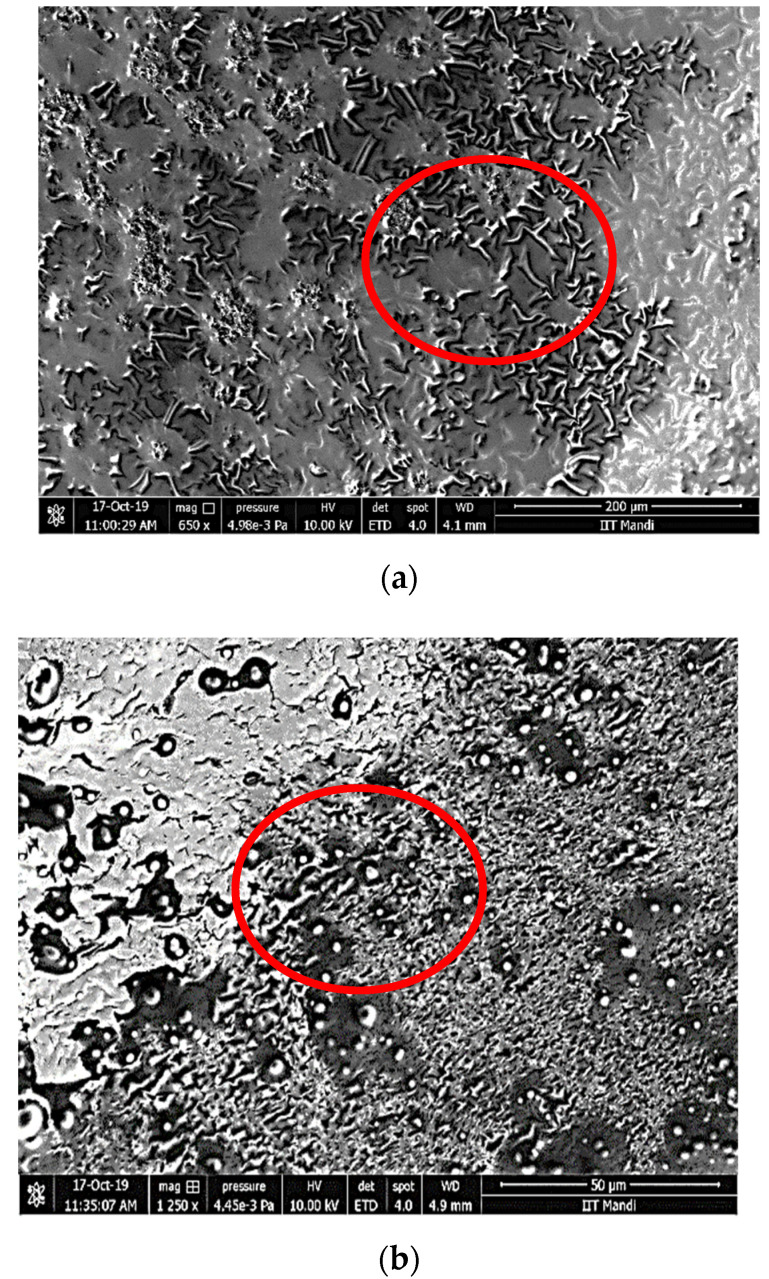
(**a**) Micrographs of control (650×) and (**b**) RRE5 (1250×) nanoemulsions indicating the smooth type and spherical nanoemulsion droplets. The presence of larger and smaller droplets in inconsequential proportions is described in different regions of the sample, which is common with nanoemulsions.

Scanning electron microscopy was used to assess the morphology of the control and RRE5 nanoemulsion samples and the results are represented in Figure 3. Both the nanoemulsions samples were of the smooth type and spherical nanoemulsion droplets visualized during scanning electron microscopy. The presence of larger and smaller droplets in inconsequential proportions can be described in different regions of the control and RRE5 nanoemulsion, which is common with nanoemulsions [36]. Our results were well supported by the findings of [34,37] who reported smooth and spherical nanoemulsion droplets after evaluating the sample using scanning electron microscopy.

### 3.4. Thermal Stability of Nanoemulsion

**Figure 4:**
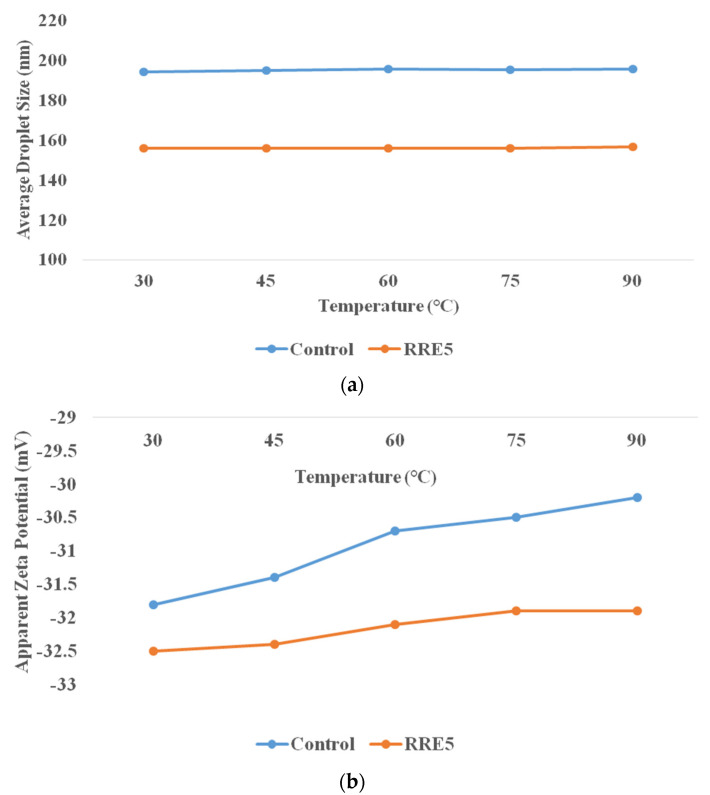
(**a**) Effect of temperature on average droplet size, (**b**) effect of temperature on zeta potential. Data are presented as means ± SD (*n* = 3).

Thermal stability was characterized to predict the effect of processing temperature on food matrixes or packages containing nanoemulsions, in order to ensure stability during final consumption [38]. The effect of thermal processing on control and RRE5 nanoemulsion is depicted in Figure 4a,b. Both control and RRE5 nanoemulsions showed significant differences from each other during thermal stability evaluation. RRE5 nanoemulsion showed significantly (*p* < 0.05) higher thermal stability in comparison with the control nanoemulsion. A non-significant (*p* < 0.05) difference was observed in the average droplet size with an increasing temperature range. Moreover, no visual creaming or oiling off was observed during the thermal processing of the RRE5 nanoemulsion. Similarly, the non-significant difference in the zeta potential of RRE5 was observed with increasing temperature. Gum arabic forms the thin interfacial layers, therefore, the range of steric repulsion generated by Tween 80 and gum arabic was not long enough, hence, the nanoemulsion droplets were stabilized against aggregation. Furthermore, interfacial tension in RRE5 nanoemulsion is also related to the influence of Laplace pressure, in which low values facilitate the breakage of droplets, generating small average droplet sizes and higher stability against coalescence with increasing temperature [39].

### 3.5. The Influence of pH on Nanoemulsion Formation

**Figure 5:**
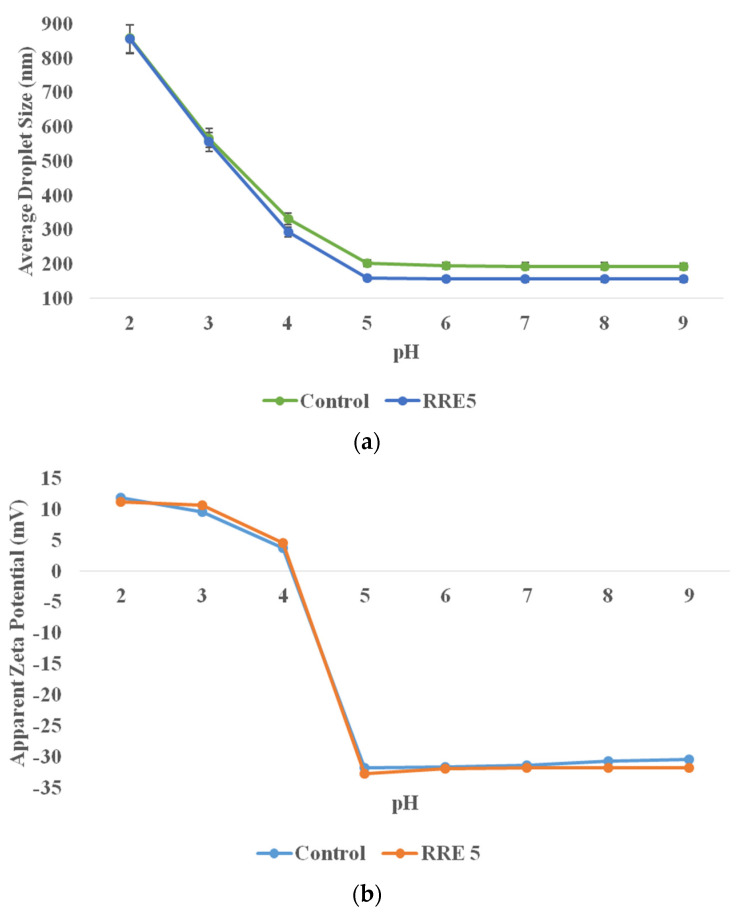
(**a**) Effect of pH on average droplet size, and (**b**) effect of pH on apparent zeta potential of RRE5 nanoemulsion. Data are presented as means ± SD (*n* = 3).

The influence of pH on droplet size and zeta potential of control and RRE5 nanoemulsion is presented in Figure 5a,b. RRE5 nanoemulsion showed significantly (*p* < 0.05) higher stability at different pH ranges as compared to the control nanoemulsion sample. At pH 5–9, the RRE5 nanoemulsion showed significantly (*p* < 0.05) smaller droplets as compared to pH 2–4 and was stable to creaming and oiling off. However, at pH 4, the nanoemulsion showed a relatively lower droplet size (294.28 nm), but a thin layer of oil was observed on the surface of the RRE5 nanoemulsion. Despite the small droplet size, some coalescence was observed. At pH 2 and 3, RRE5 nanoemulsion was highly unstable to phase separation. These facts can further be comprehended by the appearance of creaming because of the merging of oil phase droplets due to insufficient cover by gum arabic. This result was likely due to the exposure of amino groups from gum arabic molecules with an increased degree of protonation, probably caused by the demulsification [36].

### 3.6. The influence of Ionic Strength on Nanoemulsion Formation

**Figure 6:**
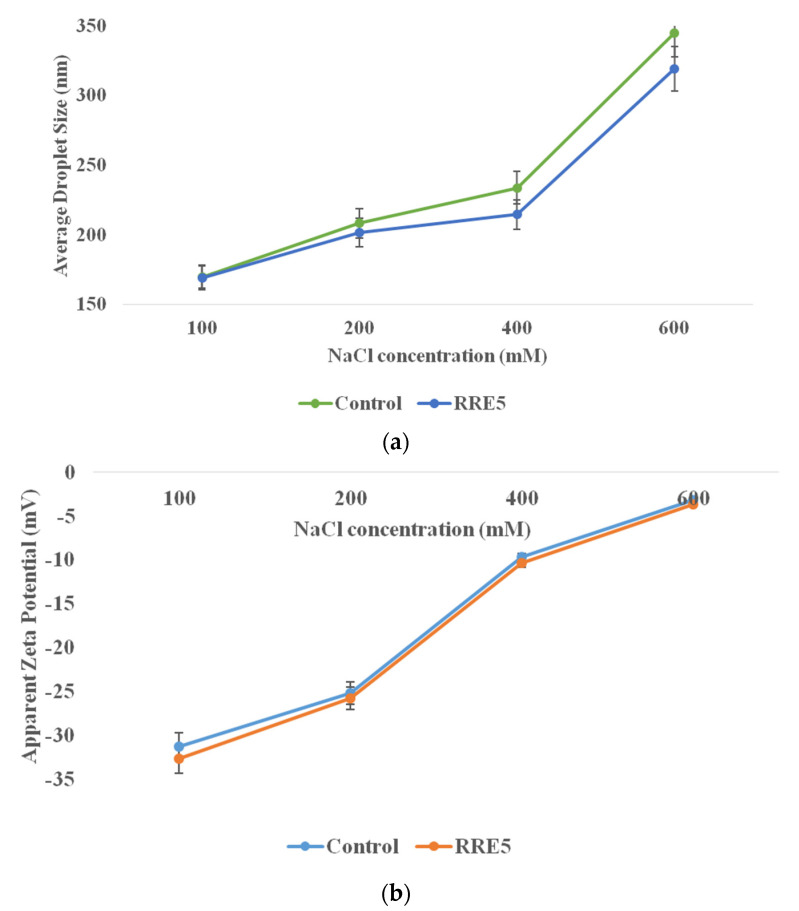
(**a**) Effect of NaCl concentration on average droplet size, and (**b**) effect of NaCl concentration on apparent zeta potential of RRE5 nanoemulsion. Data are presented as means ± SD (*n* = 3).

The effect of ionic strength on droplet size and zeta potential of control and RRE5 nanoemulsion is represented in Figure 6a,b. RRE5 nanoemulsion showed significantly (*p* < 0.05) improved ionic strength as compared to the control nanoemulsion sample. Moreover, RRE5 nanoemulsion showed a significant (*p* < 0.05) increase in average droplet size with an increasing concentration of NaCl. Columbic repulsion and negatively charged carboxylate of gum arabic reveal the polyanionic structure of gum arabic. This structure led to electrostatic interaction between the negative charge and positive charge of sodium ions, hence, increased droplet size was observed. In addition, the electrostatic interaction also affected the zeta potential of the RRE5 nanoemulsion and exerted a significant (*p* < 0.05) decrease in the zeta potential value. The presence of protein moiety in gum arabic structure interacted with negatively charged chloride ions and that interaction led to zero value of zeta potential [32]. Interestingly, the colloidal stability of nanoemulsion did not affect due to increased salt concentration [24].

### 3.7. TBA Value

**Figure 7:**
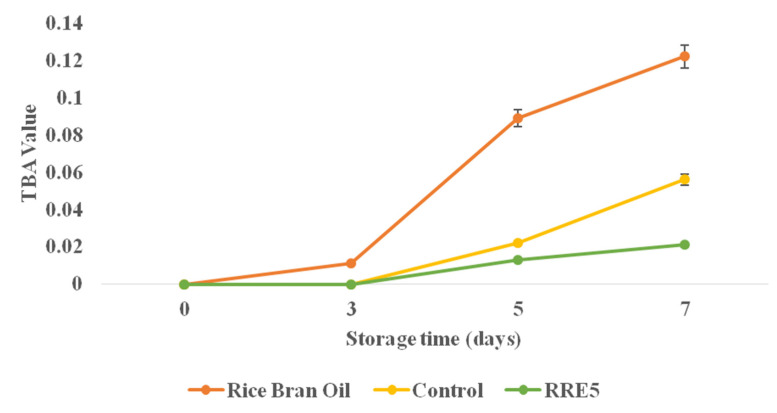
Effect of storage period (7 days) on TBA value of rice bran oil, control, and RRE5 nanoemulsion. Data are presented as means ± SD (*n* = 3).

The TBA value of control and RRE5 nanoemulsion in comparison with rice bran oil is represented in Figure 7. The TBA value for rice bran oil ranged from 0.00 to 0.12, while 0.00 to 0.002 was observed for RRE5 nanoemulsion. However, RRE5 and control nanoemulsion samples showed non-significant (*p* < 0.05) differences from each other. A significant (*p* < 0.05) difference was observed in the TBA value of rice bran oil and nanoemulsion on the 3rd, 5th, and 7th days of storage, but a non-significant difference was observed in the TBA value on 0 days for both the samples. The presence of red rice extract in the emulsion system was the possible reason for the higher oxidative stability of the RRE5 nanoemulsion [27,32]. Results are well supported by our previous findings [38] where gum arabic stabilized *Rhododendron arboretum* flower extract nanoemulsion showed significantly higher oxidative stability against free fatty acid.

### 3.8. Antioxidant Activity

**Figure 8:**
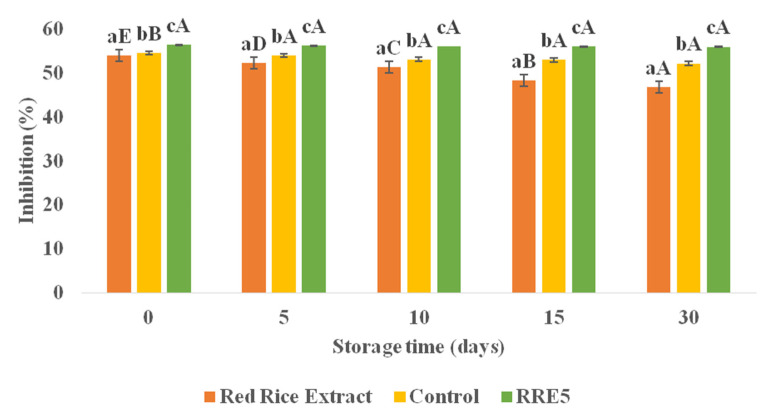
Effect of storage period (30 days) on antioxidant activity of red rice extract, control, and RRE5 nanoemulsion. Data are presented as means ± SD (*n* = 3). ^a–c^ Means within the column with different lowercase superscripts are significantly different (*p* < 0.05) from each other. ^A–E^ Means within the column with different uppercase superscripts are significantly different (*p* < 0.05) from each other.

DPPH scavenging assay was used for the evaluation of the antioxidant activity of stored emulsion sample and results of % inhibition of DPPH by control and RRE5 nanoemulsion sample in comparison with red rice extract is represented in Figure 8. The RRE5 nanoemulsion showed a significantly (*p* < 0.05) higher percentage inhibition of DPPH as compared to the control nanoemulsion and rice extract up to 30 days of storage. Moreover, rice extract showed a significant (*p* < 0.05) decrease in percentage inhibition (54.23–49.78%) on the 10th, 15th, and 30th days of storage, but rice extract showed a non-significant (*p* < 0.05) difference in percentage inhibition on the 5th day of storage. However, on the other hand, control and RRE5 nanoemulsions showed a non-significant (*p* < 0.05) difference in percentage inhibition (56.32–55.89%) throughout the storage period. The explanation may rely on the kinetic release of the antioxidants from the oil core. Various intrinsic bioactive components are known to have first-order release kinetics from nanoemulsions similar to the oxidation rates observed for *Rhododendron arboretum* and *Ficus palmata* loaded nanoemulsions [35,38]. Moreover, results of oxidative stability of nanoemulsion also support the trend of antioxidant activity of RRE5 nanoemulsion.

### 3.9. Anti-Inflammatory Activity

**Figure 9:**
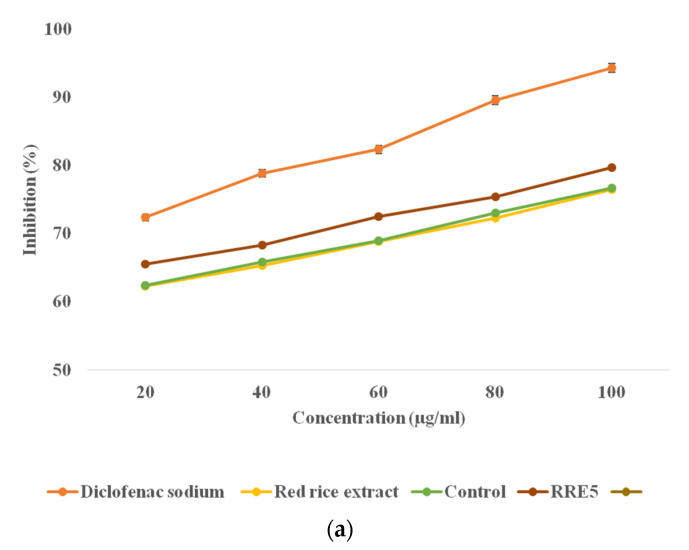

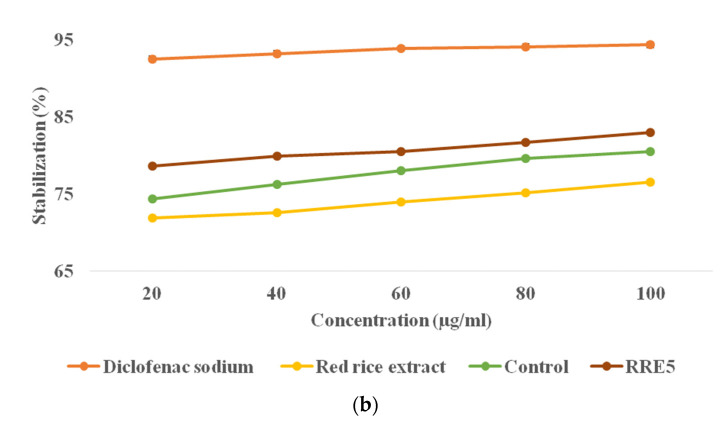
In vitro anti-inflammatory efficiency of red rice extract, control, and RRE5 nanoemulsion by (**a**) protein denaturation and (**b**) HRBC membrane stabilization process. Data are presented as means ± SD (*n* = 3).

A protein (BSA) denaturation assay and HRBC membrane stabilization assay were used for the in vitro anti-inflammatory assay and the results are represented in Figure 9a,b. During membrane stabilization and protein, a denaturation assay significant (*p* < 0.05) difference was observed in the anti-inflammatory activity of red rice extract, control nanoemulsion, and RRE5 nanoemulsion. Red rice extract, control, and RRE5 nanoemulsion were able to inhibit the protein denaturation and stabilized the HRBC membrane in a concentration-dependent manner (20–100 µg/mL). RRE5 revealed a significantly (*p* < 0.05) higher inhibition of protein denaturation (66.39–75.79%) and membrane stabilization (65.56–75.79%) in comparison with control nanoemulsion and red rice extract (65.17–72.64%) and (62.36–76.47%) with increasing concentration, respectively. However, diclofenac sodium salt showed significantly (*p* < 0.05) higher percentage stabilization and percentage inhibition in comparison with rice extract and RRE5 emulsion. The formation of the metabolic component during the in vitro assay led to the decreased anti-inflammatory activity of red rice as compared to RRE5 nanoemulsion. Furthermore, the emulsification technique enhanced the functionality of red rice extract in the context of inhibition of BSA denaturation. Inflammation and oxidation are closely related and free radicals that damage the cells lead to inflammation. It is a preventive effort by an organism for removing injurious stimuli alongside inflammation to start a signal for the healing process. The evidence proves that membrane stabilization is an additional mechanism of the anti-inflammatory effect of the samples. The release of lysosomal contents from neutrophils might be inhibited at the inflammation sites for membrane stabilization [40]. Results are in agreement with the beneficial effects of oil in water nanoemulsions for the oral administration of drugs [34,41]. Further, it could be pointed out here that it is the synergistic influence of both droplet size and the rice bran oil presence in a continuous phase of the emulsion that offers the effective anti-inflammatory activity of red rice extract.

### 3.10. Antimicrobial Efficiency

**Figure 10:**
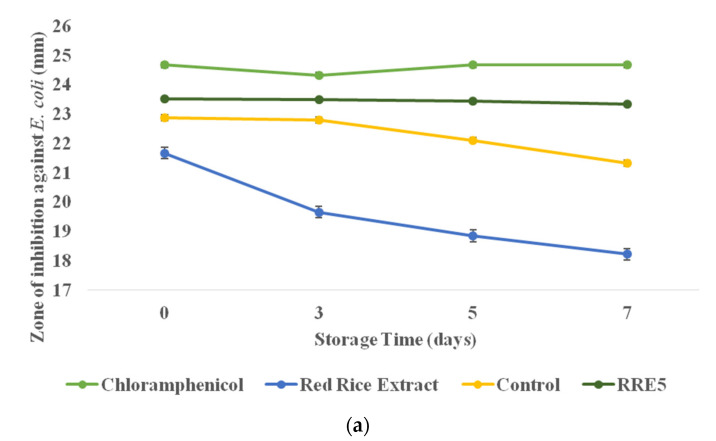

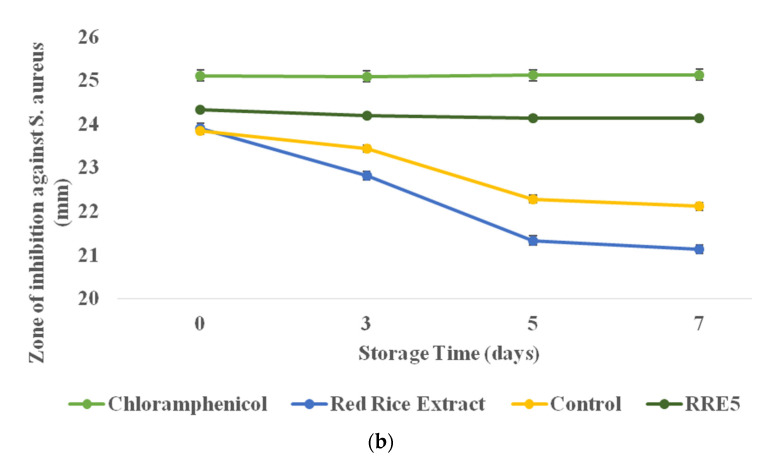
Effect of storage time (7 days) on the antimicrobial efficacy of red rice extract, control, and RRE5 nanoemulsion against (**a**) *Escherichia coli*, (**b**) *Staphylococcus aureus.* Data are presented as means ± SD (*n* = 3).

The antimicrobial properties of RRE5 nanoemulsion in comparison with red rice extract were evaluated against both Gram-positive and Gram-negative bacterial strains. The obtained diameter as the zone of inhibition (mm) is depicted in Figure 10a,b. Herein, both red rice extract and nanoemulsion were stored at 37 °C for 7 days and the antimicrobial activity of both samples was evaluated during storage. In the case of Gram-negative bacteria, RRE5 nanoemulsion showed a significantly (*p* < 0.05) higher zone of inhibition in comparison with red rice extract; however, a non-significant difference was observed as compared to positive control in terms of zone of inhibition. Furthermore, red rice extract showed significantly (*p* < 0.05) less antimicrobial activity against *Escherichia coli* during 7 days of storage as the zone of inhibition was decreased from 21.77 to 16.33 mm. However, on the 5th and 7th day of storage, a non-significant (*p* < 0.05) difference was observed. Whereas RRE5 nanoemulsion did not show any decrease in the zone of inhibition during storage, in the case of Gram-positive bacteria, nanoemulsion showed a significantly (*p* < 0.05) higher zone of inhibition than that of red rice extract.

Moreover, the RRE5 nanoemulsion showed a non-significant (*p* < 0.05) difference in the zone of inhibition during 7 days of storage, but it showed significantly (*p* < 0.05) higher antimicrobial efficiency as compared to the control nanoemulsion. Red rice extract showed a significant (*p* < 0.05) decrease in the zone of inhibition during storage. Therefore, from the results, it can be concluded that due to temperature, the antimicrobial efficacy of red rice was decreased, but RRE5 showed promising results in terms of antimicrobial properties. Here, RRE5 nanoemulsion showed significantly (*p* < 0.05) fewer antimicrobial properties against *Escherichia coli* as compared to *Staphylococcus aureus.* According to the literature, the sensitivity of the *Staphylococcus aureus* in comparison with *Escherichia coli* can be attributed to the membrane-bounded periplasm that is present in its cell membrane (consisting of peptidoglycan composed of techoic and teichuronic acid). Furthermore, the outer cell wall of *S. aureus* is a formulation of a thick hydrophobic structure in which a large number of proteins and lipids can bind. This porous membrane could be the reason for the increased permeability of RRE5 extract [42]. On the other hand, in *E. coli*, the outer membrane consists of a protective layer of phospholipids bound to the inner leaflets and lipopolysaccharides bound to the outer leaflets. The alteration in these structures including mutation in porins, changes in hydrophobic properties, and other factors may result in the resistance of bacteria towards chemotherapeutic and naturally occurring bioactive components [30,43,44]. Results of the time-kill study of red rice extract and RRE5 nanoemulsions are presented in Table 1 and Table 2. Here, a similar dilution, i.e., 1.5 × 10^8^ was used to evaluate the killing efficiency of the samples with increasing time intervals. Moreover, in the case of *Escherichia coli*, RRE5 nanoemulsion showed a significantly (*p* < 0.05) lower (7.85 Log CFU/mL) value as compared to control nanoemulsion (7.91 Log CFU/mL) and red rice extract (7.97 Log CFU/mL), but a non-significant (*p* < 0.05) difference was observed until 18 h of incubation. Similarly, in the case of *Staphylococcus aureus,* the RRE5 nanoemulsion showed a significantly (*p* < 0.05) lower (7.79 Log CFU/mL) value as compared to the control nanoemulsion (7.84 Log CFU/mL) red rice extract (8.05 Log CFU/mL), but a non-significant (*p* < 0.05) difference was observed until 18 h of incubation. The results of the time-kill study were well supported by the antimicrobial potential of the RRE5 nanoemulsion.

## 4. Conclusions

Environmental stress on plant extracts always leads to less bioactivity and generation of unwanted metabolites. Hence, to overcome this problem and to increase the functionality of bioactivity of red rice extract, gum arabic stabilized nanoemulsions were formulated. Among all samples, RRE5 nanoemulsion was selected for stability testing, antioxidant activity, anti-inflammatory, and anti-microbial activity. Over a different range of temperatures, pH, and salt concentrations, RRE5 nanoemulsion showed higher stability to creaming or flocculation. Moreover, nanoemulsion improved the oxidative stability of rice bran oil and red rice extract during storage. Furthermore, RRE5 showed significantly (*p* < 0.05) enhanced antioxidant and anti-inflammatory activity. Nanoemulsion samples also revealed more effective antimicrobial activity against Gram-positive and negative bacteria than that of red rice extract. In conclusion, gum arabic stabilized nanoemulsion has shown great potential for antioxidant and anti-inflammatory activity. Therefore, it could be used as an effective food preservative and potential anti-inflammatory and antimicrobial agent.

## Figures and Tables

**Table 1:** Time-kill Study of red rice extract, control, and RRE5 nanoemulsion against *Escherichia coli*.

Time (h)	Red Rice Extract (Log CFU/mL)	Control Nanoemulsion (Log CFU/mL)	RRE5 Nanoemulsion (Log CFU/mL)
0	8.40 ± 0.29 ^a^	8.39 ± 0.42 ^a^	8.38 ± 0.37 ^a^
18	8.29 ± 0.34 ^a^	8.28 ± 0.22 ^a^	8.26 ± 0.59 ^a^
24	8.05 ± 0.27 ^c^	7.95 ± 0.55 ^b^	7.92 ± 0.24 ^a^
48	7.99 ± 0.31 ^c^	7.91 ± 0.59 ^b^	7.85 ± 0.33 ^a^

Data are presented as means ± SD (*n* = 3). ^a–c^ Means within the column with different lowercase superscripts are significantly different (*p* < 0.05) from each other.

**Table 2:** Time-kill study of red rice extract and RRE5 nanoemulsion against *Staphylococcus aureus*.

Time (h)	Red rice Extract (Log CFU/mL)	Control Nanoemulsion (Log CFU/mL)	RRE5 Nanoemulsion (Log CFU/mL)
0	8.26 ± 0.36 ^a^	8.27 ± 0.19 ^a^	8.25 ± 0.39 ^a^
18	8.18 ± 0.43 ^a^	8.17 ± 0.38 ^a^	8.15 ± 0.32 ^a^
24	7.97 ± 0.23 ^c^	7.93 ± 0.51 ^b^	7.87 ± 0.26 ^a^
48	7.89 ± 0.44 ^c^	7.84 ± 0.47 ^b^	7.79 ± 0.41 ^a^

Data are presented as means ± SD (*n* = 3). ^a–c^ Means within the column with different lowercase superscripts are significantly different (*p* < 0.05) from each other.

## Data Availability

Data sharing is not applicable to this article.

## Abbreviations

**DLS**	Dynamic light scattering
**DPPH**	2,2-diphenyl-1-picrylhydrazyl
**RRE**	Red rice extract
**GA**	Gum arabic
**TBA**	2-thiobarbituric acid
**BSA**	Bovine serum albumin
